# Retroperitoneal Bronchogenic Cyst Mimicking Hydatid Liver: A Case Report

**DOI:** 10.1155/2012/312147

**Published:** 2012-02-01

**Authors:** Fazl Q. Parray, Afak Yusuf Sherwani, Sajad Ahmad Dangroo, Rafia Aziz Bisati, Nighat Shaffi Malik

**Affiliations:** Department of Surgery, Sher-i-Kashmir Institute of Medical Sciences, Soura, Jammu and Kashmir, Srinagar 190011, India

## Abstract

Bronchogenic cysts frequently occur in the mediastinum. They may be rarely encountered in the abdomen and retroperitoneum. Bronchogenic cysts can in fact mimic hydatid cysts. We report a case of retroperitoneal bronchogenic cyst below the right hemidiaphragm mimicking a hydatid cyst of the liver in a 30-year-old female.

## 1. Introduction

Bronchogenic cysts are rare benign congenital anomalies that result from abnormal budding of the developing tracheobronchial tree, with separation of the buds from the normal airways [1]. Bronchogenic cysts are lined by respiratory epithelium with bronchial glands, smooth muscle, and cartilage. Although bronchogenic cysts frequently occur in the mediastinum, they may be rarely encountered in the abdomen and retroperitoneum [1, 2]. Most of the reported cases have been diagnosed incidentally [1, 2]. Bronchogenic cysts are usually asymptomatic, unless they become secondarily infected or enlarge enough to compress adjacent organs or may perforate [2]. A case of adenocarcinoma arising in a retroperitoneal bronchogenic cyst has also been reported in literature [3].

## 2. Case Report

**Figure 1:**
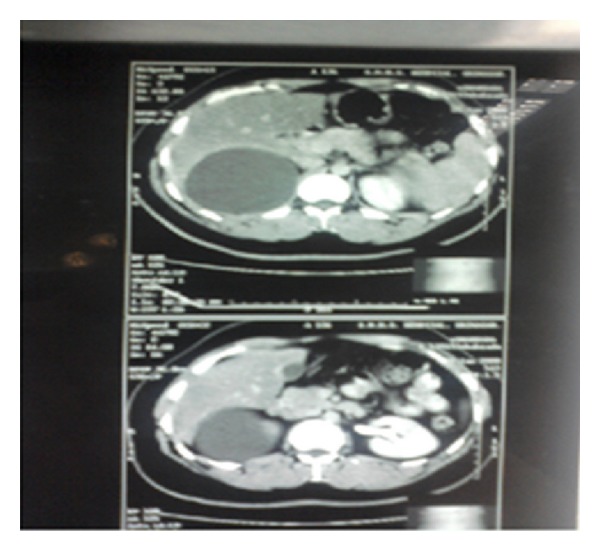


**Figure 2:**
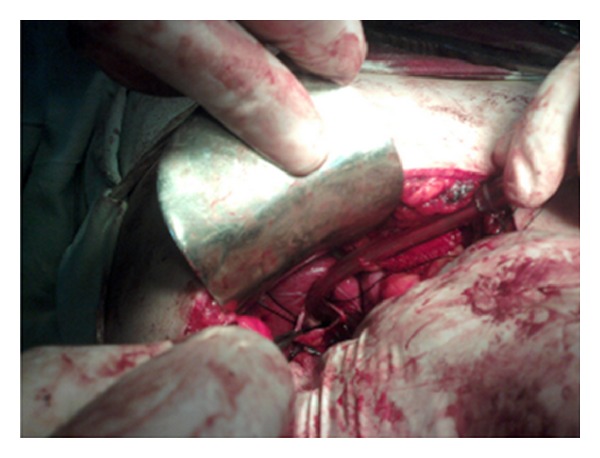


**Figure 3:**
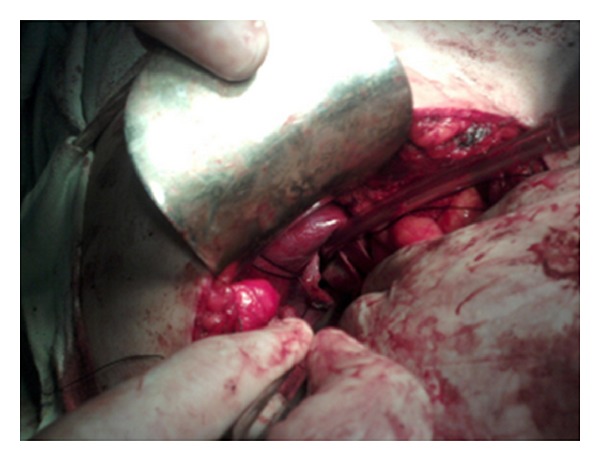


 A 30-year-old female was admitted in our department with chief complaints of intermittent pain right upper abdomen of 4-year duration. Past history was insignificant, and systemic examination was normal. Complete blood counts, liver and kidney function tests were within normal limits. ELISA for hydatid serology was negative. Ultrasonography abdomen revealed a cystic area 340 cubic centimeters in volume in relation to segment 6 of liver and extending to right suprarenal region. Contrast computerized tomographic scan of the abdomen was done which revealed a unilocular cyst in the right lobe of liver with a provisional diagnosis of hydatid cyst liver (Figure 1). Exploratory laparotomy revealed a normal appearing liver with a 10 × 8 × 8 centimetres cyst in the retroperitoneum between liver and right suprarenal area and extending to the right hemidiaphragm (Figures 2 and 3). Intraoperatively, the cyst was located only after liberal mobilization of liver and suprarenal area, the cyst was located quite deep in retroperitoneum in the immediate vicinity of inferior vena cava and portal vein. Only an indentation on the liver surface adjacent to the cystic lesion was observed. The cyst contained mutinous fluid. No evidence of laminated hydatid membrane or daughter cysts was found. Whole of the cyst fluid was drained, and the cyst was left there keeping in view the difficult location. Tube drain was put in to drain the cavity. The patient had an uneventful postoperative recovery and was discharged on 3rd postoperative day. The drain was removed after 2 weeks of followup.

## 3. Discussion

Most bronchogenic cysts originate in the mediastinum, while 15% to 20% occur in the lung parenchyma [4–6]. They can occurs in many atypical locations, ranging from the neck to the spinal dura mater, to below the diaphragm [6–8]. Bronchogenic cysts appear as spherical or oval masses with smooth outlines and are usually unilocular and noncalcified [5, 6]. Bronchogenic cysts can in fact mimic hydatid cysts. However, the CT density reading may be higher, comparable to that of soft tissue, which can create other problems in diagnosis [8]. At CT, bronchogenic cysts manifest as rounded, well-circumscribed hypoattenuating cysts without enhancement [1]. If bronchogenic cysts manifest as retroperitoneal masses, they are usually located at the subdiaphragmatic space [1, 2]. They can be misdiagnosed as solid masses because they appear hyperattenuating owing to the protein contents of the lesion [2]. In addition, bronchogenic cysts may have calcifications [2]. Although most are asymptomatic, excision is recommended to establish the diagnosis, alleviate symptoms, and to prevent complications, such as infections and the remote, but documented risk of malignant transformation [3].

 Although the occurrence of retroperitoneal bronchogenic cyst is rare, it should be considered in the differential diagnosis of a cystic lesion in the region adjacent to the liver.

 To the best of our knowledge, only 22 retroperitoneal cases have been reported in the world literature by the year 2001, 17 of which are English language reports [9].

## References

[1] Siegelman ES, Birnbaum BA, Rosato EF, Buckley JA (1998). Bronchogenic cyst appearing as a retroperitoneal mass. *American Journal of Roentgenology*.

[2] Murakami R, Machida M, Kobayashi Y, Ogura J, Ichikawa T, Kumazaki T (2000). Retroperitoneal bronchogenic cyst: CT and MR imaging. *Abdominal Imaging*.

[3] Sullivan SM, Okada S, Kudo M, Ebihara Y (1999). A retroperitoneal bronchogenic cyst with malignant change. *Pathology International*.

[4] Rapado F, Bennett JD, Stringfellow JM (1998). Bronchogenic cyst: an unusual cause of lump in the neck. *Journal of Laryngology and Otology*.

[5] St-Georges R, Deslauriers J, Duranceau A (1991). Clinical spectrum of bronchogenic cysts of the mediastinum and lung in the adult. *Annals of Thoracic Surgery*.

[6] Di Lorenzo M, Collin PP, Vaillancourt R, Duranceau A (1989). Bronchogenic cysts. *Journal of Pediatric Surgery*.

[7] Suen HC, Mathisen DJ, Grillo HC (1993). Surgical management and radiological characteristics of bronchogenic cysts. *Annals of Thoracic Surgery*.

[8] Ramenofsky LM, Leape LL, McCauley RG (1979). Bronchogenic cyst. *Journal of Pediatric Surgery*.

[9] Haddadin WJ, Reid R, Jindal RM (2001). A retroperitoneal bronchogenic cyst: a rare cause of a mass in the adrenal region. *Journal of Clinical Pathology*.

